# JUNB suppresses distant metastasis by influencing the initial metastatic stage

**DOI:** 10.1007/s10585-021-10108-9

**Published:** 2021-07-19

**Authors:** Juliane Wutschka, Bettina Kast, Melanie Sator-Schmitt, Sila Appak-Baskoy, Jochen Hess, Hans-Peter Sinn, Peter Angel, Marina Schorpp-Kistner

**Affiliations:** 1 Division of Signal Transduction and Growth Control, DKFZ-ZMBH Alliance, Im Neuenheimer Feld 280, 69120 Heidelberg, Germany; 2 Faculty of Biosciences, University Heidelberg, Heidelberg, Germany; 3 Department of Otorhinolaryngology, Head and Neck Surgery, Heidelberg University Hospital, Heidelberg, Germany; 4 Research Group Molecular Mechanisms of Head and Neck Tumors, DKFZ, Heidelberg, Germany; 5 Institute of Pathology, University of Heidelberg, Heidelberg, Germany; 6 Present Address: Department of Chemistry and Biology, Ryerson University, Toronto, ON Canada; 7 Present Address: iBEST (Institute of Biomedical Engineering, Science and Technology), Toronto, ON Canada

**Keywords:** Metastasis, Transcription factor, Tumor microenvironment

## Abstract

The complex interactions between cells of the tumor microenvironment and cancer cells are considered a major determinant of cancer progression and metastasis. Yet, our understanding of the mechanisms of metastatic disease is not sufficient to successfully treat patients with advanced-stage cancer. JUNB is a member of the AP-1 transcription factor family shown to be frequently deregulated in human cancer and associated with invasion and metastasis. A strikingly high stromal JUNB expression in human breast cancer samples prompted us to functionally investigate the consequences of JUNB loss in cells of the tumor microenvironment on cancer progression and metastasis in mice. To adequately mimic the clinical situation, we applied a syngeneic spontaneous breast cancer metastasis model followed by primary tumor resection and identified stromal JUNB as a potent suppressor of distant metastasis. Comprehensive characterization of the JUNB-deficient tumor microenvironment revealed a strong influx of myeloid cells into primary breast tumors and lungs at early metastatic stage. In these infiltrating neutrophils, BV8 and MMP9, proteins promoting angiogenesis and tissue remodeling, were specifically upregulated in a JUNB-dependent manner. Taken together, we established stromal JUNB as a strong suppressor of distant metastasis. Consequently, therapeutic strategies targeting AP-1 should be carefully designed not to interfere with stromal JUNB expression as this may be detrimental for cancer patients.

**Supplementary Information:**

The online version contains supplementary material available at 10.1007/s10585-021-10108-9.

## Introduction

Although metastasis, the spread of tumor cells from the primary tumor to a distant organ site, is highly inefficient with less than 0.02% of disseminated tumor cells being able to establish a metastatic nodule [1], it remains a major challenge in cancer therapy. Over the last years, the tumor microenvironment as a vital determinant of tumor progression and metastasis has attracted more and more attention. It is now widely accepted that tumor cells actively remodel the stromal compartment not only at the primary tumor but also at the distant organ site. This distant site remodeling takes place prior to any evidence of metastatic disease, generates a more permissive environment and, thus, facilitates metastatic outgrowth. The formation of this so-called “pre-metastatic niche” [2] by various tumor-derived factors and extracellular vesicles [3] involves the attraction of various immune cells, vascular remodeling and induction of angiogenesis, metabolic reprogramming and alterations in the extracellular matrix composition (reviewed in [4–6]).

The Activating Protein-1 family member JUNB has been proven to govern multiple processes linked to pre-metastatic niche formation. Particularly in the vasculature, JUNB is of uttermost importance. In mice, global *Junb* deficiency results in embryonic lethality due to failure in placental and embryonic neovascularization [7]. Furthermore, JUNB is required for full induction of *Vegfa* upon hypoxia, tumor angiogenesis [8], and vascular contractility and motility by regulation of myosin regulatory light chain 9 [9]. In zebrafish, Junb impacts the development of the lymphatic vasculature via its direct target miR-182 [10]. Besides vascular remodeling, JUNB deficiency promotes inflammation by acting as a suppressor of multiple pro-inflammatory cytokines and chemokines, such as *lipocalin-2* [11], *Gm-csf* [12], *Cxcl1* [12] and *Cxcl2* [12] and regulates diverse processes contributing to immunosurveillance and immune evasion in multiple immune cell types. Macrophage activation as well as CD4+ T cell polarization to T_H_2 cells depend on JUNB-mediated expression of *Il-1β* [13] and *Il-4* plus *Infγ* [14, 15], respectively. In cancer, JUNB is frequently deregulated and dependent on the cancer entity can act as tumor suppressor [16–19] or oncogene [20–22]. Multiple studies have associated JUNB with invasion and metastasis. Knockdown of *Junb* in tumor cells suppressed invasiveness in renal-cell carcinoma [23] and breast cancer [24], and *Junb* knockout reduced metastasis in an experimental metastasis model of head and neck squamous cell carcinoma [25]. In contrast, JUNB levels were negatively correlated to tumor stage and lymph node involvement in breast cancer specimens [26]. Nevertheless, these studies have not addressed the vital importance of the tumor microenvironment and neglected the complexity of the metastatic cascade.

In this study, we, thus, aimed to investigate the consequences of *Junb* deletion in stromal cells for metastasis, particularly for early metastatic stages. For this purpose, we applied a spontaneous breast cancer metastasis model to conditional *Junb* knockout mice and studied angiogenesis as well as immune cell infiltration. Here, we show that JUNB acts as a suppressor of distant metastasis, which is paralled by diminished immune cell infiltration at the initial metastatic stage.

## Materials and methods

### Tissue microarray

Human tissue microarray samples (0.6 mm cores) were obtained from the National Center for Tumor Disease (Institute of Pathology, University Hospital Heidelberg, Germany) and stained for JUNB (C37F9, CST, 1:500) by immunohistochemistry as previously described [27]. Imaging was performed on the Axio Scan.Z1 (Zeiss, Germany) and raw image files were adjusted for brightness, contrast and gamma using the Zen Blue software (Zeiss). Further details in Online Resource data and Table S1.

### Cells

EO771 and EO771.LMB-mCherry cells [28] were obtained from Robin L. Anderson (Peter MacCallum Cancer Centre, Melbourne). Cells were maintained as previously described [28] and were free from Epstein-Barr virus, Squirrel-Monkey Retrovirus, mycoplasma and cross-contaminating cells from species other than mouse (Multiplexion, Heidelberg, Germany). Cells were regularly checked for mycoplasma contamination (Minerva biolabs®, Berlin, Germany). EO771-GFP cells were generated by lentiviral transduction using the pLVTHM vector (Addgene, Watertown, MA, USA).

### Mice

Animal care and all animal experiments were performed according to the national guidelines and approved by the local government authorities of the state Baden-Württemberg (Regierungspräsidium Karlsruhe, authorization numbers G-206/13, G-26/16, G-93/18, G-227/18).

The generation of conditional *Junb* knockout mice *Junb*^Δ/Δ^ had been described previously [12]. In brief, hemizygous *Junb* knockout mice (*Junb*^Δ/+^; generated by previous mating to CMV-Cre Deleter mice) were crossed with Col1α2-Cre mice, where Cre expression is driven by the Collagen type (I)-alpha2 promoter [29] starting at E8.5–9.5. Col1α2-Cre-positive *Junb*^Δ/+^males were crossed to *Junb*^flox/flox^ female mice [30]. As controls *Junb*^+/+, Col1α2-Cre^ mice were used. For fibroblast-specific tamoxifen-inducible *Junb* knockout mice, *Junb*^flox/flox^ mice were bred to Col1α2-CreER (T) [31] transgenic mice, *Junb*^+/+, Col1α2-CreER(T)^ were used as controls. Deletion of *Junb* was induced by Tamoxifen injection (1 mg/100 µL, Sigma-Aldrich, Steinheim, Germany) i.p. on 5 consecutive days before the start of tumor experiments. All mice were on a C57BL/6N background (F > 12) and used for experiments at the age of 6–8 weeks.

### Animal experiments

The spontaneous metastasis experiment was essentially performed as previously described [28]. For tumor induction, 1 × 10^5^ EO771.LMB-mCherry cells were injected into the 4th mammary fat pad on the left. Tumor size was regularly measured using a digital caliper and volume was determined using the formula V = 1/2 × length × width^2^. For spontaneous metastasis experiments, tumors were excised at approximately 500 mm^3^ and mice were sacrificed 21 days after. General well-being of the mice was regularly monitored and mice were given analgesia with 2 mg/kg of bodyweight Meloxicam s.c. (Boehringer Ingelheim, Biberach, Germany) pre- and postoperatively. Early metastatic lungs were harvested at the time point of resection.

For the experimental metastasis assay, 5 × 10^5^ EO771.LMB-mCherry or EO771-GFP were injected into the tail vein. Mice were euthanized 19 days after injection and organs were dissected for analysis. Mice injected with EO771-GFP were additionally sham-operated over the 4th mammary fat pad on the left 1 day after tail vein injection. These mice received analgesia with 2 mg/kg of bodyweight Meloxicam s.c.

Organs were collected after perfusion with PBS through the left ventricle either in 700 µL QIAzol® lysis reagent (Qiagen, Hilden, Germany) and immediately snap-frozen in liquid nitrogen for subsequent DNA/RNA isolation or transferred to 4% paraformaldehyde/PBS for histological analyses. For analysis of lung metastasis, the left superior lung lobe was used for DNA/RNA isolation whereas the remaining lung was used for histology.

### Flow cytometric analysis

Depending on the panel, dead cell discrimination with a fixable viability dye was performed prior to surface marker staining. After washing in PBS, a maximum of 1 × 10^6^ cells was stained with freshly reconstituted fixable viability dye at 1 µL/1 mL PBS for 30 min. Excess dye was removed by washing in PBS. Unspecific binding to Fc-receptors was blocked by resuspending in anti-CD16/CD32 antibody (clone 93, ebioscience, 1:100). After 5–10 min incubation appropriate antibody cocktails for surface marker staining (respective panels in Online Resource data) were added 2× concentrated and cells were stained for 20min on ice.

Prior to acquisition, cells were passed through a 37 µm filter and viability marker 7-AAD (Becton Dickinson, Erembodegem, Belgium) was added at 3 µL/1 × 10^6^ cells if required. Samples were analyzed either at a BD FACSCanto II or a BD LSRFortessa (BD Biosciences, San Jose, CA, USA). Cell sorting of neutrophils and macrophages was performed at a BD FACSAria1 machine (BD Biosciences) and cells were sorted into FACS buffer. Data were analyzed using FlowJo v10 (Tree Star, Inc., Ashland, OR, USA).

### RNA isolation

Tissues harvested in QIAzol® lysis reagent were homogenized using an ULTRA-TURRAX® T25 (IKA® Labortechnik, Staufen, Germany) and subjected to RNA isolation using the miRNeasy® Mini Kit (Qiagen) according to manufacturer’s instructions. For RNA isolation of MACS-purified neutrophils the miRNeasy® Micro Kit (Qiagen) was used.

On-column DNA digestion was performed with the RNase-Free DNase Set (Qiagen) following the manufacturer’s manual.

Cell pellets obtained after centrifugation of FACSorted cells were resuspended in 100 µL Extraction buffer of the Arcturus® PicoPure® RNA Isolation Kit (Thermo Fisher Scientific, Vilnius, Lithuania) and incubated for 30min at 42 °C. The obtained cell extract was briefly spun down and stored at − 80 °C. RNA isolation was performed following the manufacturer’s instructions including on-column treatment with DNase I (Qiagen).

Quality and quantity of RNA were assessed using the NanoDrop 1000 system (PeqLab Biotechnology, Erlangen, Germany) and stored at − 80 °C until further use.

### DNA isolation

According to Ambion’s ToTALLY RNA^TM^ RNA Isolation Kit protocol (Thermo Fisher) remaining organic phases after chloroform extraction for RNA isolation were combined with an equal volume of 10 mM Tris-HCl, 0.1M NaCl, 1mM EDTA, 1%SDS, pH12 and mixed well. After 10 min incubation on ice, samples were centrifuged at 12000×*g*, 4 °C for 20min. To the aqueous phase, 1/15 volume of 7.5 M NH_4_OAc and 2 volumes of 100% ethanol were added and, after inverting, the sample was stored at − 20 °C O/N. DNA was pelleted by centrifugation at 12000×*g*, 4 °C for 30min, washed in 70% ethanol and finally dissolved in DNase-free water. After determination of DNA quality and quantity using the NanoDrop 1000 system, DNA concentration was adjusted to 20 µg/µL and stored at − 20 °C.

### Quantitative real-time PCR

cDNA was generated from up to 2 µg total RNA using RevertAid Reverse Transcriptase (200 U/μL, Thermo Fisher Scientific). Quantitative real-time PCR was performed using Power SYBR^TM^ Green Master Mix (Applied Biosystems, Woolston, UK) on the StepOnePlus Real-Time PCR system (Applied Biosystems) with cDNA corresponding to 2.5ng of RNA per reaction as input. Respective primers used for qPCR are listed in Table S2 of the Online Resource.

Metastatic load was assessed by quantification of the *mCherry* or *Gfp* reporter gene present in EO771.LMB-mCherry and EO771-GFP cells, respectively, on total DNA isolated from metastatic lungs as described before [28, 32] with small adaptations: samples were analyzed with the Power SYBR^TM^ Green assay on the StepOnePlus Real-Time PCR system (Applied Biosystems).

### Immunohistochemistry/immunofluorescence

Sections were deparaffinized, rehydrated and subsequently subjected to antigen retrieval depending on the primary antibody (Online Resource Table S3). After blocking with 10% normal goat serum (Vector Laboratories, Burlingame, CA, USA) sections were incubated with primary antibodies at 4 °C O/N. For colorimetric signal detection, sections were treated with biotinylated secondary antibodies (1:500, Vector Laboratories) after washing with PBS followed by immersion in 3% H_2_O_2_/methanol to block endogenous peroxidases. Signal was amplified with the VECTASTAIN® Elite® ABC HRP Kit (Vector Laboratories) and subsequently visualized using DAB Peroxidase Substrate Kit (Vector Laboratories). Finally, sections were counterstained and mounted as described in Online Resource.

For immunofluorescent detection, sections were treated with fluorescently-labelled secondary antibodies (1:250, Thermo Fisher Scientific) and nuclei were stained with Hoechst 33342 (1:1000, chemodex, Worksop, UK). Slides were mounted using Fluorescent mounting medium (Dako, Carpintiera, CA, USA). For detection of murine JUNB, slides were immersed in 0.03% Triton-X100/PBS after deparaffinization and rehydration and heated to 60 °C for 15min to allow penetration of the nuclear envelope. Slides were then allowed to cool down to RT in the same buffer, subjected to antigen retrieval and incubated with anti-JUNB (C37F9, CST, Leiden, Netherlands, 1:200) at 4 °C O/N. Biotinylated secondary anti-rabbit antibody (1:500, Vector Laboratories) followed by AlexaFluor^®^647-labelled streptavidin (1:500, Jackson Immunoresearch, Ely, UK) was utilized for signal amplification and fluorescent detection.

Immunofluorescent co-staining of JUNB and CD31 was performed on cryo-sections. For this purpose, paraformaldehyde-fixed tissues (24 h) were equilibrated in 30% sucrose/PBS and embedded in Tissue-Tek®O.C.T.™ Compound (Sakura,Torrance, CA, USA) thereafter. After air-drying the sections for 5 min and quick rinsing in PBS, staining was performed as described above.

Images were acquired on the Nikon Eclipse Ti (Nikon, Düsseldorf, Germany) or Axio Scan.Z1 (Zeiss, Oberkochen, Germany). For presentation, raw images were processed and adjusted for brightness, contrast and gamma using the Zen Blue software (Zeiss) or FIJI (Image J, National Institutes of Health, USA). Unedited images were quantified utilizing macros for FIJI (Image J) written by Damir Krunic.

Lung metastatic burden was determined by quantification of immunohistochemical staining for mCherry or GFP. Whole lung had been sectioned and images of 6–7 whole lung sections were taken using the 10x objective of the Axio Scan.Z1.

### Statistical analysis

All statistical tests performed in this study are indicated in the respective figure legend, p < 0.05 was considered statistically significant. Normally distributed data were subjected to two-sided unpaired t-test, data not following a normal distribution, such as relative gene expressions and metastatic burden, were analyzed by Mann-Whitney test. Data were analyzed and visualized using GraphPad Prism 7.05 (Graphpad Software, Inc., La Jolla, CA, USA).

Further details on experimental procedures can be found in the Online Resource.

## Results

### JUNB is highly expressed in human mammary carcinoma

**Fig. 1:**
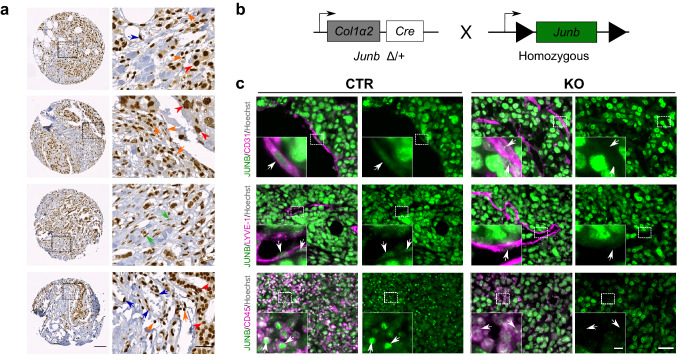
JUNB expression in human and murine breast cancer tissue. **a** JUNB expression in tumor cells (red arrowhead), endothelial cells (blue arrow), immune cells (orange arrow) and fibroblasts (green arrow). Scale bar in overview 100 µm, picture on the right shows magnification of the area marked in overview, scale bar in magnification 30 µm. **b** Schematic representation of *Junb* knockout (KO) mice. **c** Immunofluorescence co-staining of EO771.LMB primary tumors of JUNB (green) with CD31 (pink, first panel, endothelial cells), LYVE-1 (pink, second panel, lymphatics) and CD45 (pink, last panel, immune cells); Hoechst (grey) marks nuclei. Scale bar 30 µm, in magnification 5 µm

To assess JUNB levels, we stained a tissue microarray of human mammary carcinoma for JUNB by immunohistochemistry. In the majority of evaluable specimen we found JUNB to be highly expressed in tumor cells independent of clinicopathological parameters (Online Resource Fig. 1a–d and Table S1). Yet, we also noticed strong JUNB positivity in cells of the surrounding stroma, such as endothelial cells, fibroblasts and immune cells (Fig. 1a). As JUNB levels are tightly controlled and JUNB is hardly expressed in adjacent non-tumor tissue [26, 33, 34], we wondered whether this high stromal JUNB expression might impact the metastastic spread. For this purpose, we utilized a well-established conditional *Junb* knockout mouse (*Junb*^Δ/Δ Col1α2Cre^, named JUNB KO [9, 12, 35]. Here, *Junb* deletion occurs not only in mesenchyme-derived but rather all stromal cells including fibroblasts, endothelial cells, pericytes and immune cells [12, 35] due to very prominent Col1α2-driven Cre expression in fibroblasts [29] as well as very low level Cre expression in cells during embryonic development resulting in *Junb* deletion due to the permissive chromatin of this immediate early gene.

These JUNB KO mice were compared to transgenic mice being wildtype for *Junb* but carrying the Col1α2-Cre transgene (named *Junb*^+/+^, CTR) (Fig. 1b and c). Indeed, immunofluorescent co-stainings of JUNB and cell type specific markers, revealed the absence of JUNB specifically in blood (CD31+) and lymphatic (LYVE-1+) endothelial cells as well as immune cells (CD45+) of KO but not in the CTR mice (Fig. 1c; Online Resource Fig. 1e). Furthermore, JUNB was highly expressed in EO771.LMB tumor cells (Fig. 1c; Online Resource Fig. 1e). Thus, we could use this so far only known C57BL/6-mouse-derived model of spontaneously metastatic mammary cancer [28] to analyze the contribution of stromal JUNB to metastasis.

### Stromal JUNB loss promotes distant metastasis

**Fig. 2:**
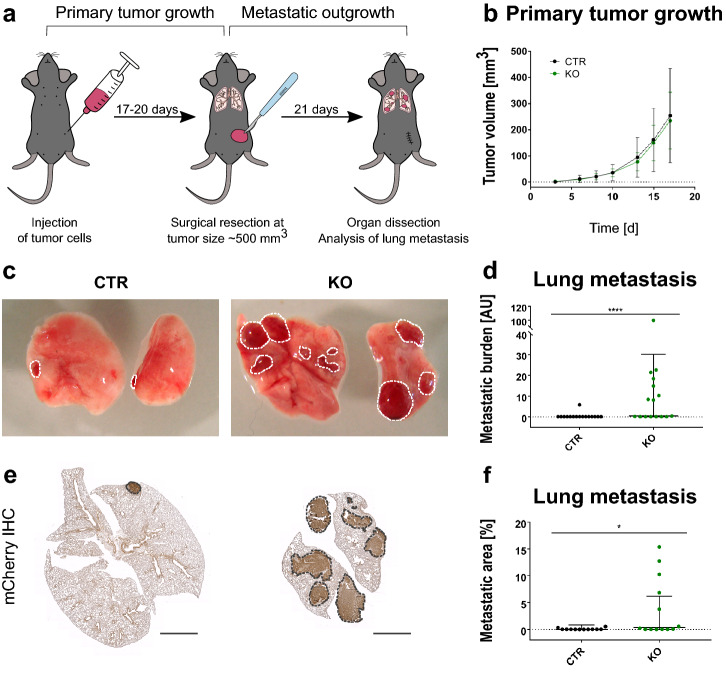
Loss of JUNB promotes distant metastasis. **a** Schematic representation of the spontaneous metastasis assay using EO771.LMB tumor cells. **b** Primary tumor growth of EO771.LMB in CTR and KO mice. Data points represent the mean, error bars SD of all mice of the respective genotype, n = 15 (CTR) and n = 16 (KO). **c** Macroscopic images of lung metastasis. **d** Quantification of lung metastatic burden by qPCR of the *mCherry* reporter compared to *vimentin* on DNA level. Mann Whitney test p < 0.0001. n = 16 per group. **e** Representative images of the immunohistochemistry (IHC) for mCherry, scale bar 2 mm, and the quantification thereof (**f**), n = 12 (CTR) and 13 (KO). Mann Whitney analysis revealed a significant increase of lung metastasis upon deletion of stromal *Junb*, p = 0.0257. Data in (**d**) and (**f**) are displayed as geometric mean with geometric SD, one data point represents one mouse, p. *p < 0.05, ****p < 0.0001

In order to investigate the functional consequences of stromal JUNB loss on distant metastasis, EO771.LMB breast cancer cells tagged with a mCherry reporter [28] were orthotopically injected into the mammary fat pad of *Junb* KO and CTR mice and metastasis was analyzed after surgical removal of the primary tumor (Fig. 2a). In line with our previous study [35] primary tumor growth was not affected by the absence of JUNB in the tumor stroma (Fig. 2b). Yet, when macroscopically examining the lungs at the experimental endpoint, we did observe higher metastasis in KO mice (Fig. 2c). This impression was further confirmed by both evaluating the presence of *mCherry* on genomic DNA level (Fig. 2d) as well as on protein level by immunohistochemical staining (Fig. 2e and f).

### Loss of JUNB is associated with elevated immune cell recruitment at the early metastatic stage

**Fig. 3:**
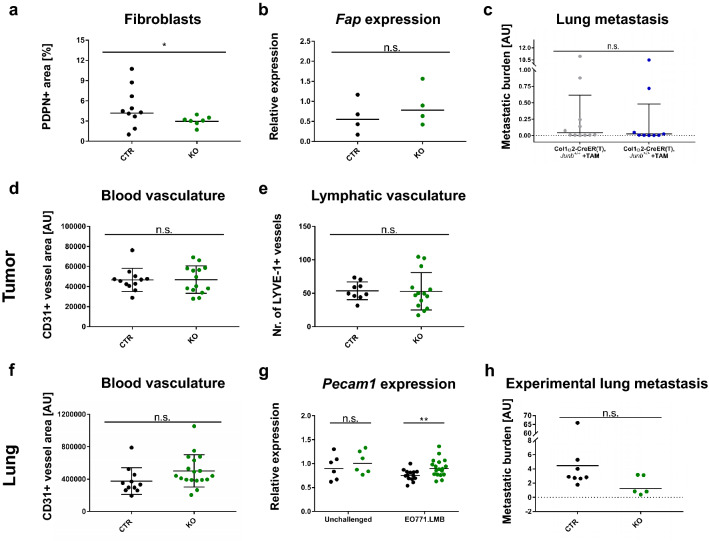
JUNB does not alter fibroblast density or tumor vasculature. **a**, **b** Quantification of fibroblast content in primary tumors by immunohistochemistry for Podoplanin (**a**; PDPN), n = 10 (CTR) and n = 7 (KO), Mann Whitney analysis: p = 0.0431, and gene expression analysis of *fibroblast activating protein* (B; FAP), n = 4 for both CTR and KO, Mann Whitney: p = 0.6857. **c** Distant lung metastasis in conditional fibroblast-specific mice as quantified via the presence of the *mCherry* reporter in tumor cells in whole genomic DNA, n = 11 (Col1α2-CreER(T), *Junb*^+/+^, + TAM) and n = 9 (Col1α2-CreER(T), *Junb*^>/>^, + TAM), data obtained from three independent injection rounds, Mann Whitney: p = 0.4119. **d** Tumor blood vasculature as assessed by CD31 immunofluorescence staining. Quantification was based on two whole tumor sections with 5 random fields each. Necrotic areas were avoided. Significance was assessed by unpaired t-test, p = 0.9521, n = 12 (CTR) and n = 14 (KO). **e** Number of lymphatic vessels in primary tumors, LYVE-1 + structures were manually counted on two whole tumor sections, n = 9 (CTR), n = 14 (KO). Unpaired t-test, p = 0.9449. **f** Blood vasculature in early metastatic lungs quantified by immunofluorescence staining for CD31, n = 11 (CTR), n = 18 (KO), unpaired t-test: p = 0.0895. **g** Gene expression analysis of *Pecam1* in whole lungs of unchallenged and tumor-bearing mice, n = 6 (unchallenged CTR and KO), n = 14 (tumor-bearing CTR), n = 18 (tumor-bearing KO). Mann Whitney analysis of lungs from unchallenged (p = 0.3939) and tumor-bearing mice (p = 0.0079). **h** Experimental lung metastasis of EO771.LMB-mCherry cells as quantified by qPCR of the *mCherry* reporter on DNA level, n = 8 (CTR) and n = 5 (KO) from two independent injection rounds. Significance assessed by Mann Whitney test, p = 0.1709. *p < 0.05, **p < 0.01. Data are shown as means and SD in (**d**–**f**), in (**a**, **b**, ** g**, **h**) the geometric mean and in (**c**) the geometric mean plus geometric SD are indicated. Data points represent individual mice

As fibroblasts represent a major component of the tumor microenvironment, we first analyzed the fibroblast content in EO771.LMB primary tumors. Fibroblast density was slightly, but significantly reduced when assessed by immunohistochemistry for podoplanin (PDPN, Fig. 3a, Online Resource Fig. 2c) but no change was observed measuring gene expression of fibroblast activating protein (FAP, Fig. 3b). Importantly, fibroblast-specific deletion of *Junb* using a tamoxifen-inducible Col1α2-CreER(T) did not affect distant metastasis excluding a cell-intrinsic fibroblast-specific effect (Fig. 3c).

In the past, our group had established JUNB as an essential regulator of neovascularization [7], angiogenesis [8] and lymphatic development [10] prompting us to investigate whether a defective vasculature may be the cause of enhanced metastatic spread. Analysis of CD31+ blood and LYVE-1+ lymphatic vascular density did not reveal any JUNB-dependent changes in EO771.LMB primary tumors (Fig. 3d and e, Online Resource Fig. 2a and b). Assessment of the blood vascular area in lungs at the initial metastatic stage by both immunofluorescence staining and gene expression analysis using the indicative vessel marker *Pecam1* revealed a slight increase upon loss of *Junb* (Fig. 3f and g), yet, a similar tendency was also observed in naive mice (Fig. 3g). To test the integrity of the vasculature, we performed experimental metastasis assays with two different cell lines (EO771.LMB-mCherry or EO771-GFP). Intravenous injection of tumor cells did not result in enhanced metastasis in JUNB KO mice when compared to CTR mice but rather showed an opposite tendency (Fig. 3h, Online Resource Fig. 2d–g). Hence, the vasculature of JUNB KO mice is intact.

**Fig. 4:**
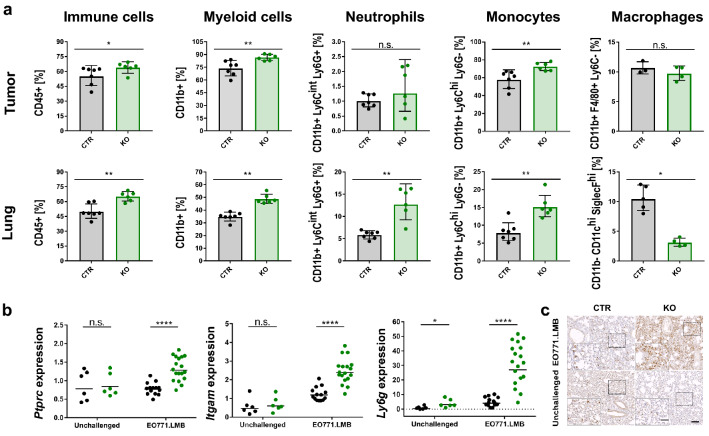
Characterization of immune landscape. **a** Increased immune cell infiltration upon *Junb* loss in EO771.LMB primary tumors and early metastatic lungs as assessed by flow cytometry, n = 7 (CTR), n = 6 (KO), from two independent experiments, macrophage data are obtained from one experiment with n = 3 (CTR) and n = 4 (KO). CD45 + immune cells are displayed as fraction of all living cells; myeloid cells, neutrophils, monocytes and macrophages are related to mCherry- CD45 + living cells. **b** Enhanced expression of immune cell markers *Ptprc* (CD45), *Itgam* (CD11b) and *Ly6g* in whole lungs of unchallenged and EO771.LMB tumor-bearing mice, n = 6 (unchallenged CTR and KO, n = 14 (CTR EO771.LMB), n = 18 (KO EO771.LMB). **c** Representative images of neutrophil infiltration (neutrophil 7/4) in lungs of unchallenged and tumor-bearing mice, scale bar 100 µm, inset shows a magnified image of the marked area, scale bar of inset 50 µm. Each data point represents one mouse. Significance was determined by Mann Whitney analysis. *p < 0.05, **p < 0.01, ***p < 0.001, ****p < 0.0001. Heights of the bar represent geometric mean, with error bars indicating geometric SD (**a**), in (**b**) geometric mean is displayed

As the immune system plays a crucial role in cancer development and progression, we next characterized the immune landscape in CTR and JUNB KO mice by flow cytometric analysis, both in the primary tumor and early metastatic lungs (gating strategy Online Resource Fig. 3a and b). JUNB-deficient mice generally displayed an enhanced immune cell infiltration compared to CTR mice, especially in the early metastatic lungs (65.0 ± 5.0% compared to 49.9 ± 7.8% CD45+ cells) (Fig. 4a). Further immune cell profiling revealed an elevated level of myeloid cells but fewer T cells in tumors and early metastatic lungs of JUNB KO mice (Fig. 4a, Online Resource Fig. 4a). Since T cell activation as measured by Interferon γ production was not consistently impaired (Online Resource Fig. 4b and c) and also the ratio of CD4+/CD8+ T cells was not influenced by JUNB expression (Online Resource Fig. 4a), we focused on myeloid cells instead.

Monocyte (15.1 ± 3.3% vs. 7.8 ± 2.9%) but even more neutrophil populations (12.7 ± 4.6% vs. 5.8 ± 1.0%) were augmented in the early metastatic lungs of JUNB KO compared to CTR mice, whereas alveolar macrophages were drastically diminished (3.1 ± 0.8% vs. 10.4 ± 2.4%) (Fig. 4a, Online Resource Fig. 3c, gating strategy Online Resource Fig. 3a and b). In the primary tumor, neutrophil and macrophage frequencies were not altered and the change in monocyte infiltration was not as striking as in the lung. To exclude the possibility that enhanced myeloid cell infiltration in JUNB-deficient mice is not tumor-induced but rather a result of enhanced granulopoiesis [19], we investigated the expression of immune cell markers by RT-qPCR in lungs of tumor-bearing and age-matched unchallenged mice (Fig. 4b). While absence of JUNB strongly augmented the marker expression of *Ptprc* (CD45), *Itgam* (CD11b) and *Ly6g* in tumor-bearing mice, it hardly had any effect in unchallenged mice, in which only a much smaller rise in *Ly6g* expression was detected. The enhanced neutrophil infiltration especially into lungs of tumor-bearing JUNB KO mice was further confirmed by immunohistochemistry (Fig. 4c). Hence, elevated myeloid recruitment in JUNB KO mice was not caused by increased granulopoiesis but is specifically induced by the primary tumor. Taken together, JUNB-deficient myeloid cells may provide the most critical determinant for early metastasis and promote metastatic outgrowth in JUNB KO mice.

### Neutrophils express high levels of tissue remodeling factors

Neutrophils and macrophages have been described to facilitate the establishment of the pre-metastatic niche and have been assigned tumor-promoting as well as anti-tumor functions. For both subtypes, extensive studies have defined adequate markers to aid classification. To preselect relevant markers in this study, we performed gene expression profiling of circulating neutrophils in the blood isolated by MACS from tumor-bearing JUNB KO and CTR mice (Online Resource Fig. 5a). Surprisingly, a classical polarization of JUNB-deficient neutrophils towards tumor-promoting functions was not detected. Instead, genes implicated in tissue remodeling and angiogenesis, such as *Mmp9* and *Bv8*, were upregulated, whereas e.g. *Arginase 1*, a common marker for immunosuppression, was even expressed at lower levels in JUNB-deficient circulating neutrophils.

**Fig. 5:**
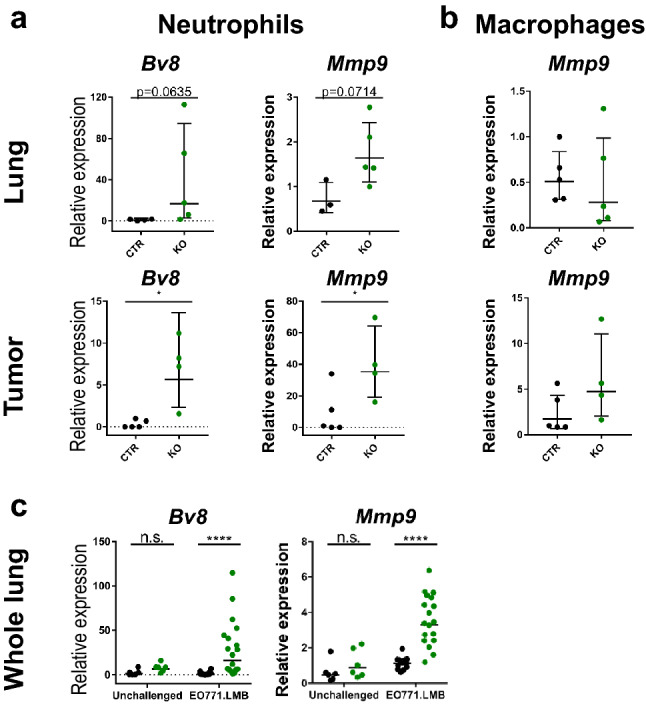
Gene expression profiling of FACSorted myeloid cells from tumor-bearing JUNB KO and CTR mice. **a** Gene expression analysis of mCherry- CD45 + CD3− B220− CD11b + Ly6G^hi^ neutrophils isolated from early metastatic lungs (upper panel) and tumors (lower panel), n = 4 (lung CTR), n = 5 (lung KO), n = 4 (tumor CTR and KO). **b** Expression of *Mmp9* in alveolar macrophages in the lung (defined as CD45 + CD3− B220− CD11b^low^ SiglecF^hi^ CD11c^hi^) and macrophages in the primary tumor (identified as mCherry− CD45 + CD3− B220− CD11b + F4/80 + Ly6C−), n = 5 (lung CTR and KO), n = 5 (tumor CTR), n = 4 (tumor KO). **c** Gene expression analysis of whole lungs from tumor-bearing (EO771.LMB) and non-tumor bearing unchallenged mice, n = 6 (unchallenged CTR + KO), n = 14 (CTR EO771.LMB), n = 18 (KO EO771.LMB). Each data point represents one individual mouse. Data are presented as geometric mean ± geometric SD (**a** + **b**) or geometric mean only (**c**). Statistical analysis (**a**–**c**) by Mann Whitney, *p < 0.05, ***p < 0.001, ****p < 0.0001

To investigate whether infiltrated myeloid cells may promote metastasis by vascular remodeling, we isolated neutrophils and macrophages from primary tumors and pre-metastatic lungs by Fluorescence-activated cell sorting (FACS) and analyzed gene expression of markers identified by screening circulating neutrophils. Both *Bv8* and *Mmp9* were expressed at a higher level in JUNB-deficient neutrophils but upregulation of *Mmp9* was neither apparent in macrophages isolated from the tumor nor alveolar or interstitial macrophages from lungs at the initial metastatic stage (Fig. 5a and b, Online Resource Fig. 5b). Analyses of whole lungs confirmed the increased expression of *Bv8* and *Mmp9* upon deletion of *Junb* specifically in tumor-bearing mice (Fig. 5c). Interestingly, elevated *Arg1* expression was observed in whole lungs upon loss of JUNB (Online Resource Fig. 5d). Although no *Arg1* upregulation was observed in isolated neutrophils or macrophages, the strong influx of neutrophils into pre-metastatic lungs may have caused overall augmented levels (Online Resource Fig. 5b). Additional markers for pro- or anti-tumor activities, such as *Il10* and *Nos2* were not found deregulated, neither in neutrophils nor macrophages (Online Resource Fig. 5b).

In order to functionally link increased neutrophil infiltration to enhanced metastatic load upon stromal deletion of *Junb*, we depleted neutrophils pharmacologically using the neutrophil depleting antibody anti-Ly6G (Online Resource Fig. 6a). Although anti-Ly6G efficiently depleted neutrophils from the blood from CTR mice, in JUNB KO mice depletion was only effective until day 10 (Online Resource Fig. 6e, gating strategy Online Resource Fig. 6f). Obviously, owing to the very high numbers of circulating neutrophils in JUNB KO mice, anti-Ly6G was not able to deplete neutrophils at later stages of tumor progression and to impair neutrophil infiltration into primary tumors and early metastatic lungs (Online Resource Fig. 6g and h). Consequently, distant metastasis was not reduced due to insufficient anti-Ly6G treatment in JUNB-deficient mice (Online Resource Fig. 6c and d). Nevertheless, this experiment confirmed the enormous increase in circulating and infiltrating neutrophils, which most likely represent the main driver for the enhanced distant metastasis upon stromal JUNB loss.

## Discussion

The importance of the pre-metastatic niche formation and the determinants for early metastatic seeding are well recognized. Yet, our understanding of the complex interplay between different cell types of the tumor microenvironment and the tumor cells is still insufficiently understood limiting the clinical success of therapies. The transcription factor JUNB had previously been linked to invasion and metastasis but studies had largely focused on tumor cells thereby ignoring the complexity of the tumor microenvironment [24, 25, 36]. Here, we describe a strong JUNB expression in cells of the tumor microenvironment of human breast cancer specimens. As JUNB levels are usually tightly controlled in physiology but often deregulated in cancer, we wondered, whether stromal JUNB may drive malignancy. Indeed, by applying a syngeneic breast cancer model of spontaneous metastasis combined with surgical excision of the primary tumor to conditional KO mice with a stromal JUNB ablation, we could identify JUNB as a strong suppressor of metastasis.

Although cancer-associated fibroblasts drive malignancy as well as invasion [37] and JUNB impacts proliferation in fibroblasts in vitro [38], we could exclude an altered fibroblast secretome as metastatic driver as fibroblast-specific deletion of JUNB did not influence distant metastasis.

Another key mediator of metastasis is a remodelled aberrant and frequently leakier vasculature promoting intravasation and extravasation of disseminated tumor cells [2, 6]. JUNB functions as an essential regulator in the blood and lymphatic vascular system determining vessel development and homeostasis [7–10]. In line with a previous study [35], in the tumor blood and lymphatic vascular density as well as primary tumor growth were not affected by JUNB loss. However, stromal JUNB loss did result in enhanced metastasis to the lung. Therefore, we reasoned that the microenvironment in the lung may be affected differently upon *Junb* deletion. Indeed, lung vascular density was higher in JUNB KO mice compared to controls, irrespective of primary tumor presence. Importantly, stromal JUNB loss did not provoke an elevated metastatic burden in two independent experimental metastasis assays. Albeit the number of animals used may appear rather small, boths assays imply that the JUNB-deficient vasculature is intact and not decisive for the observed enhanced metastasis to the lung.

Immune cells are frequently recruited to the initial metastatic site and are of vital importance in cancer progression contributing to invasion and immuno-suppression. JUNB is a key transcriptional regulator of T cell differentiation [14, 15], macrophage activation [13] as well as a well-known inhibitor of granulocyte progenitor proliferation [19]. Characterization of the immune cell landscape in JUNB-deficient mice revealed that the metastatic phenotype in stromal JUNB KO mice does not correspond to the increased anti-tumor response observed in regulatory T cell-specific JUNB knockout mice [39, 40]. Moreover, as the observed effect on T cell infiltration was visible in the primary tumor rather than the early metastatic lungs, we reasoned that enhanced metastasis is most likely not due to altered T cell infiltration and focused on myeloid cells in the tumor microenvironment instead.

These cells frequently exhibit a pro-tumorigenic phenotype, fueling immunosuppression and invasion or directly enhancing metastatic seeding. Against our expectations, gene expression analysis of isolated neutrophils and macrophages in this study did not support a clear polarization towards a pro-tumorigenic phenotype upon JUNB-deficiency. Strikingly though, markers of tissue and vascular remodeling, such as the pro-angiogenic factor *Bv8* [41] and metalloproteinase *Mmp9* [42] were significantly upregulated in JUNB-deficient neutrophils. As *Mmp9* is a known JUNB target [43–46] there is strong correlational evidence that *Mmp9* is directly suppressed by JUNB. Our hypothesis is additionally supported by ChIP-Seq data demonstrating JUNB binding to and acting as a repressor on the *MMP9* promoter in oral cancer [43]. *Bv8* could be either directly repressed by JUNB as its ortholog *Bo8* was shown to be AP-1 regulated [47] or indirectly involving a STAT3-NFkB-AP-1 transcriptional network [48, 49]. Data in the ChIP atlas data base [50] show JUNB, JUN and STAT3 binding to the murine as well as human Bv8 5’ region. Interestingly, both putative targets, *Mmp9 and Bv8*, were significantly upregulated upon stromal *Junb* loss both in the primary tumor and early metastatic lungs. While primary tumor growth is not affected, lung metastasis is enhanced. At first sight this may appear paradoxical as *Mmp9* and *Bv8* overexpression due to stromal JUNB loss should also have a positive impact on primary tumor growth. Most likely, the effect of upregulated stromal *Mmp9* and *Bv8* may be neglectable because the primary tumor itself is a rich source of pro-angiogenic and pro-inflammatory factors. By contrast, in the lungs of JUNB KO mice, the elevated influx of neutrophils in combination with the increased expression of *Mmp9 and Bv8* may culminate in an overall amplification of MMP9 and Bv8, thus, facilitating metastasis. Importantly, *Mmp9* was not upregulated in JUNB KO macrophages clearly identifying infiltrating neutrophils as a major source.

To validate the hypothesis that JUNB-deficient neutrophils promote metastasis by enhancing tissue remodeling via *Bv8* and *Mmp9* production, we aimed to deplete neutrophils pharmacologically. Yet, despite the high efficiency of anti-Ly6G antibody in the initial phase, at later stages of tumor progression depletion was technically impossible, at least with our approach, most likely due to the vast neutrophil numbers in JUNB-deficient mice. Our findings are in line with a previous report on spontaneous metastasis from 4T1 tumors in syngeneic Balb/c mice [51] showing effective neutrophil depletion only until day 14 post tumor engraftment. Admittedly, this represents a major limitation of our study but also points out that functionally defining the decisive cell type is extremely challenging and ultimately may be impossible due to the fact that JUNB represents a multifaceted transcriptional regulator of immune cells [13–15, 19]. As the EO771.LMB cell line is to our knowledge the only metastasizing breast cancer cell line derived from C57BL/6 mice, we were unfortunately unable to corroborate our findings in an additional breast cancer model. Thus, the functional link between augmented lung infiltration with JUNB-deficient neutrophils and enhanced metastasis remains to be formally established. However, neutrophils have frequently been attributed pro-metastatic functions [52, 53], also by inducing vascular remodeling [54]. We, therefore, consider it most likely that *Junb* knockout neutrophils facilitate metastasis by promoting angiogenesis and vascular remodeling in the pre-metastatic lungs. However, we cannot fully exclude a contribution of other cell types or a mechanism involving immunosuppression.

In summary, we identified JUNB as a potent suppressor of metastasis by interfering with the initial metastatic stage. As JUNB is a common downstream target of anti-proliferative cancer therapy, efforts have to be taken ensuring the design of tumor-specific therapies leaving JUNB levels in the tumor microenvironment unaffected.

## Supplementary Information

Below is the link to the electronic supplementary material.

Supplementary file1 (DOCX 4468 kb)

## Data Availability

Raw data are available from the corresponding author upon reasonable request.

## Code availability

The datasets generated and analyzed during the current study are available from the corresponding author on reasonable request.
