# Supplementary material for: JUNB suppresses distant metastasis by influencing the initial metastatic stage
Source: Clin Exp Metastasis. 2021 Jul 19;38(4):411–23. doi: 10.1007/s10585-021-10108-9 (PMC8318945; doi:10.1007/s10585-021-10108-9)
Supplement: Supplementary file 1 — Supplementary file1 (DOCX 4468 kb) [file 10585_2021_10108_MOESM1_ESM.docx]

**Supplementary data**

JUNB suppresses distant metastasis by influencing the initial metastatic stage

Juliane Wutschka^1,2^, Bettina Kast^1^, Melanie Sator-Schmitt^1^, Sila Appak-Baskoy^1,#^, Jochen Hess^3^, Hans-Peter Sinn^4^, Peter Angel^1^ and Marina Schorpp-Kistner^1^

^1^ Division of Signal Transduction and Growth Control, DKFZ-ZBMH Alliance, Heidelberg, Germany

^2^ Faculty of Biosciences, University Heidelberg, Heidelberg, Germany

^3^ Department of Otorhinolaryngology, Head and Neck Surgery, Heidelberg University Hospital, and Research Group Molecular Mechanisms of Head and Neck Tumors, DKFZ, Heidelberg, Germany.

^4^ Institute of Pathology, University of Heidelberg, Heidelberg, Germany.

^#^ present address: Ryerson University, Department of Chemistry and Biology and iBEST (Institute of Biomedical Engineering, Science and Technology), Toronto, Ontario, Canada

**Supplementary Figure legends:**

**Supplementary Fig.1**: JUNB expression in human breast cancer tumor and stromal cells evaluated by either observation (manual score) or automated analysis (H-score) of a breast cancer tissue microarray in all samples (**a**), differentiated according to hormone receptor status (**b**), tumor type (**c**) and in primary tumors which had or had not metastasized (**d**). Data are represented as mean scores ±SD (**b**-**d**). Each dot represents the mean of two cores from one respective patient sample except for two samples from metastasizing tumors that were collected from only one core. Statistical analysis by Kruskal-Wallis. *p<0.05, ***p<0.001, ****p<0.0001. IDC, invasive duct carcinoma; ILC, invasive lobular carcinoma; DIVI, ductal invasive predominantly intraductal carcinoma, others (mucinous, tubular and medullary breast cancer). **e**: Representative images of immunohistochemical staining for JUNB in CTR and JUNB KO mice. Surrounding stroma of primary tumors from JUNB high expressing (EO771.LMB) and low-expressing (B16F10) tumor cells is shown as well as lymph nodes from naïve mice, scale bar 25µm.

**Supplementary Fig. 2**: Representative immunofluorescence images of CD31+ blood endothelial vasculature (**a**) and LYVE-1+ lymphatic vessels (**b**) on EO771.LMB-mCherry primary tumors, scale bar 100 µm. **c** Fibroblast density in EO771.LMB-mCherry primary tumors as assessed by immunohistochemistry for PDPN, scale bar 100 µm. **d** Quantification of mCherry+ lung metastatic nodules in experimental metastasis assay, quantification was conducted by manually counting mCherry+ nodules, shown is the mean of results obtained from 4 whole lung sections per mouse. Individual data points represent single mice. **e** Representative images of whole lung sections from the experimental metastasis assay quantified in (**d)** and stained for mCherry by immunohistochemistry, scale bar 2 mm. **f** Metastatic burden in lung after tail vein injection of EO771-GFP followed by sham-operation as assessed by quantification of the *Gfp* reporter on DNA level. **g** Quantification of GFP+ metastatic area in the lung of these mice.

**Supplementary Fig. 3: a** Gating strategy for the analysis of immune cell populations in primary tumors and early metastatic lungs shown in Fig. 4a. **b** Panel and gating strategy for the analysis of macrophage populations shown in Fig. 4a and FACSorting of neutrophils and macrophages presented in Fig. 5. **c** Representative FACS plots of neutrophils in primary tumors and early metastatic lungs, frequencies displayed as CD11b+ Ly6G+ Ly6C^int^ of all CD45+ cells.

**Supplementary Fig.4**: **a** Infiltration of T cells into primary tumors and early metastatic lungs quantified by flow cytometry, n=7 (CTR) and n= 6 (KO), from 2 independent experiments, percentage of CD3+ in CD45+ mCherry- living cells is presented. **b** Interferon γ production in CD4+ and CD8+ T cells by flow cytometry, n=5 for CTR and KO in tumor and lung, n=4 for spleen. **c** Gating strategy for analyzing Interferon γ+ CD4+ and CD8+ T cells, representative example shown for spleen. Statistical analysis using Mann Whitney test, **p<0.01. One data point in panel **a**+**b** represents one individual mouse. In (**a**) heights of the bar represent geometric mean, error bars indicate geometric SD (A), in (**b**) data are presented as geometric mean ± geometric SD.

**Supplementary Fig. 5**: **a** Gene expression analysis by RT-qPCR of circulating neutrophils in the blood from tumor-bearing JUNB KO and CTR mice, each column represents one mouse, data are presented as ΔΔC_T_ normalized for presentation. **b** Gene expression in isolated neutrophils and macrophages from tumor and early metastatic lung, statistical analysis by Mann Whitney, p-values given in (**c**), *p<0.05. **d** *Arg1* expression in whole lungs from tumor-bearing (EO771.LMB) and non-tumor bearing unchallenged mice, n=6 (unchallenged CTR+KO), n=14 (CTR EO771.LMB), n=18 (KO EO771.LMB). Statistical significance assessed by Mann Whitney analysis, p=0.3939 (unchallenged), p=0.0007 (EO771.LMB). Each data point represents one individual mouse. Data are presented as geometric mean (**d**) and geometric mean ± geometric SD.

**Supplementary Fig. 6**: **a** Schematic presentation of the neutrophil depletion experiment. **b** Primary tumor growth of CTR mice treated with isotype control (n=5) or depleting antibody (n=6) as well as KO mice following injection of isotype control (n=4) or depleting antibody (n=5). Data are presented as mean ± SD. **c** Quantification of distant metastasis of these mice via the presence of the *mCherry* reporter on DNA level by qPCR. **d** Representative images of lungs harvested at endpoint and stained for H&E, scale bar 2 mm. **e** Neutrophil depletion efficiency evaluated by flow cytometry in blood from these mice, gating strategy shown in (**f**). Neutrophil recruitment was assessed by gene expression analysis of *Ly6g* in bulk primary tumors (**g**) and early metastatic lungs (**h**). Data in (**c)** and (**g**-**h**) are shown for individual mice, geometric mean ± geometric SD are indicated where appropriate. Bar heights in (**e**) indicate the geometric mean with error bars displaying geometric SD. Statistical analysis performed: Mann Whitney test, *p<0.05, **p<0.01.

**Supplementary Fig. 1**


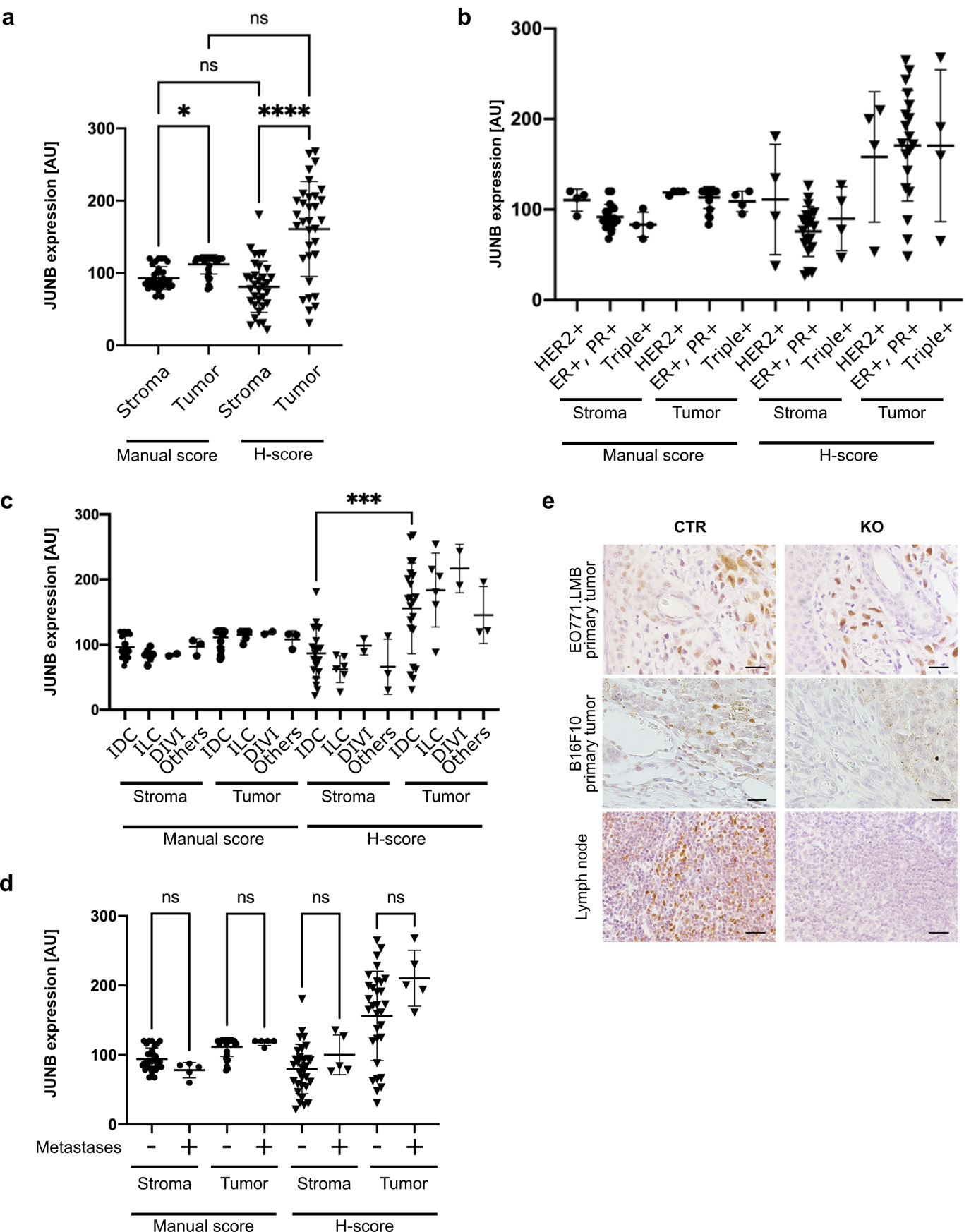


Supplementary Fig. 2

**
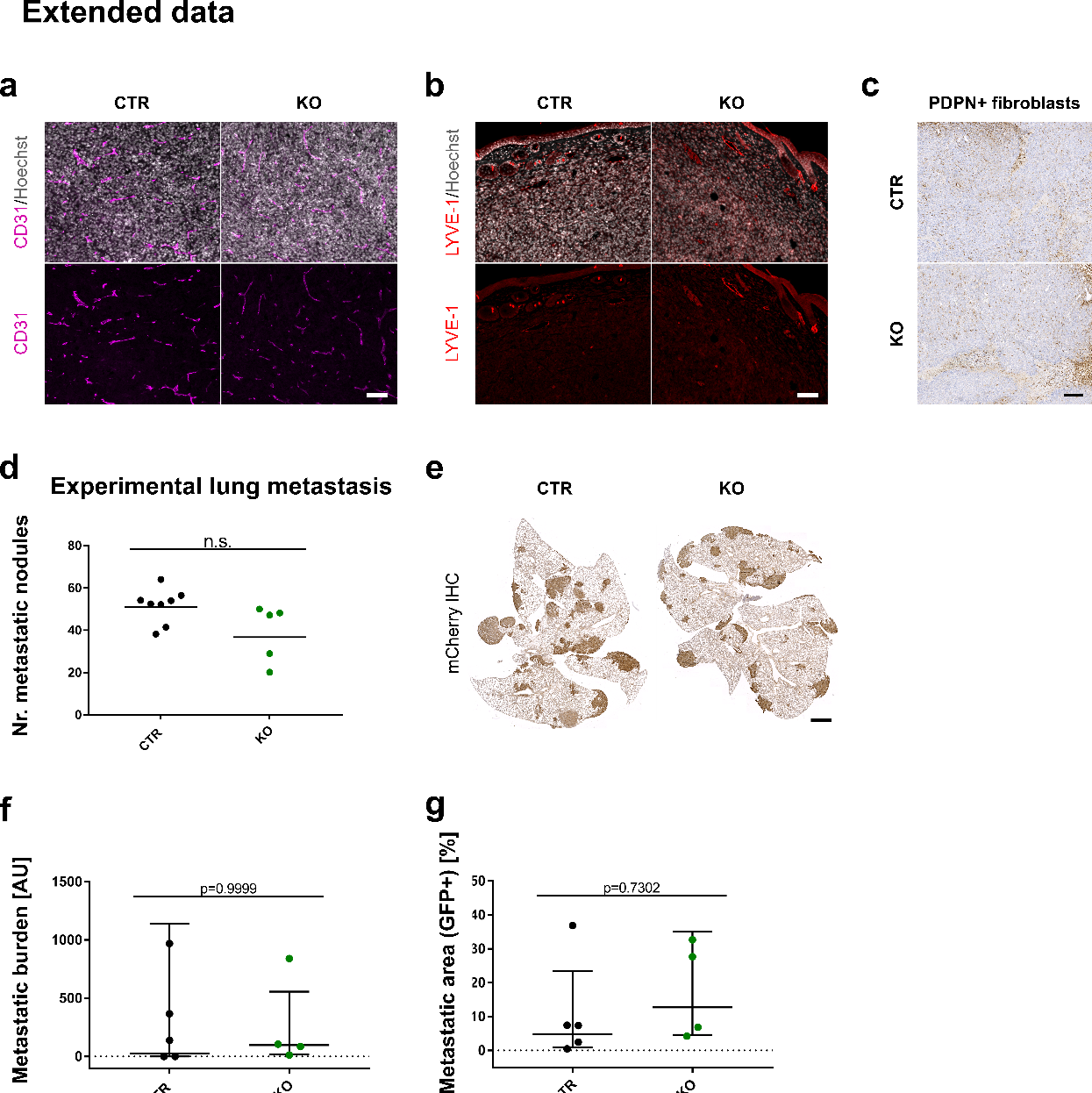
**

Supplementary Fig. 3


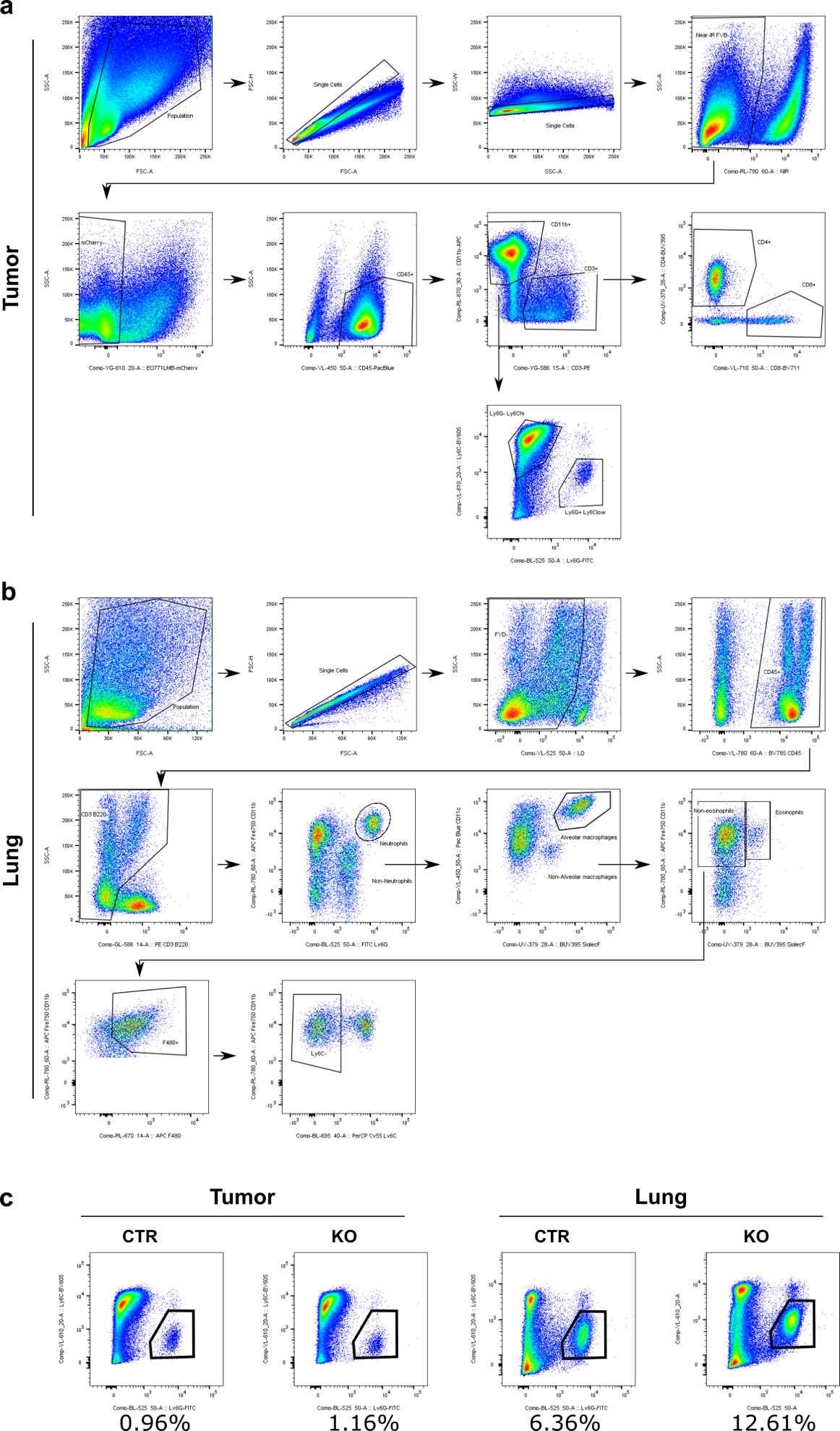


Supplementary Fig. 4


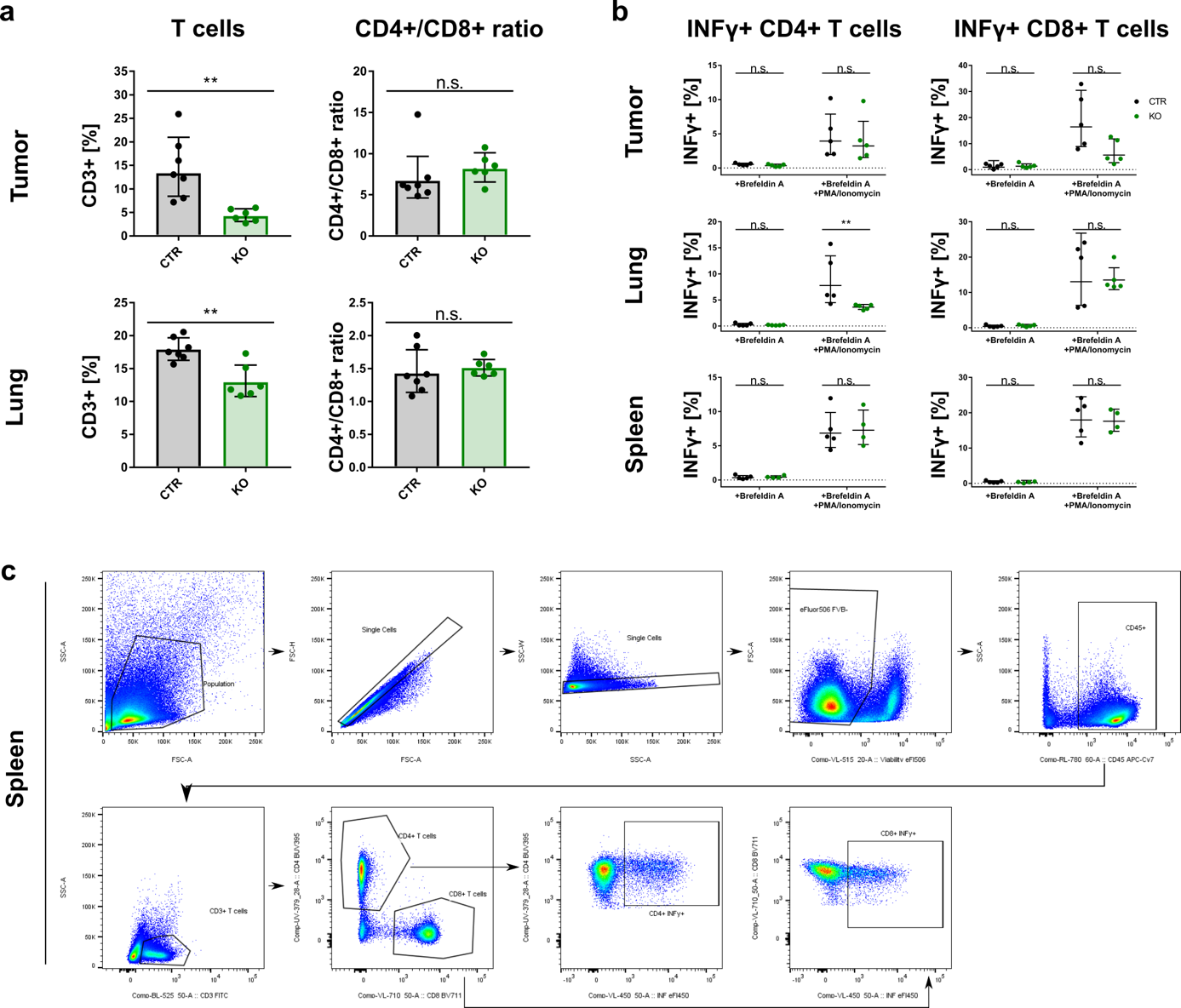


Supplementary Fig. 5

**
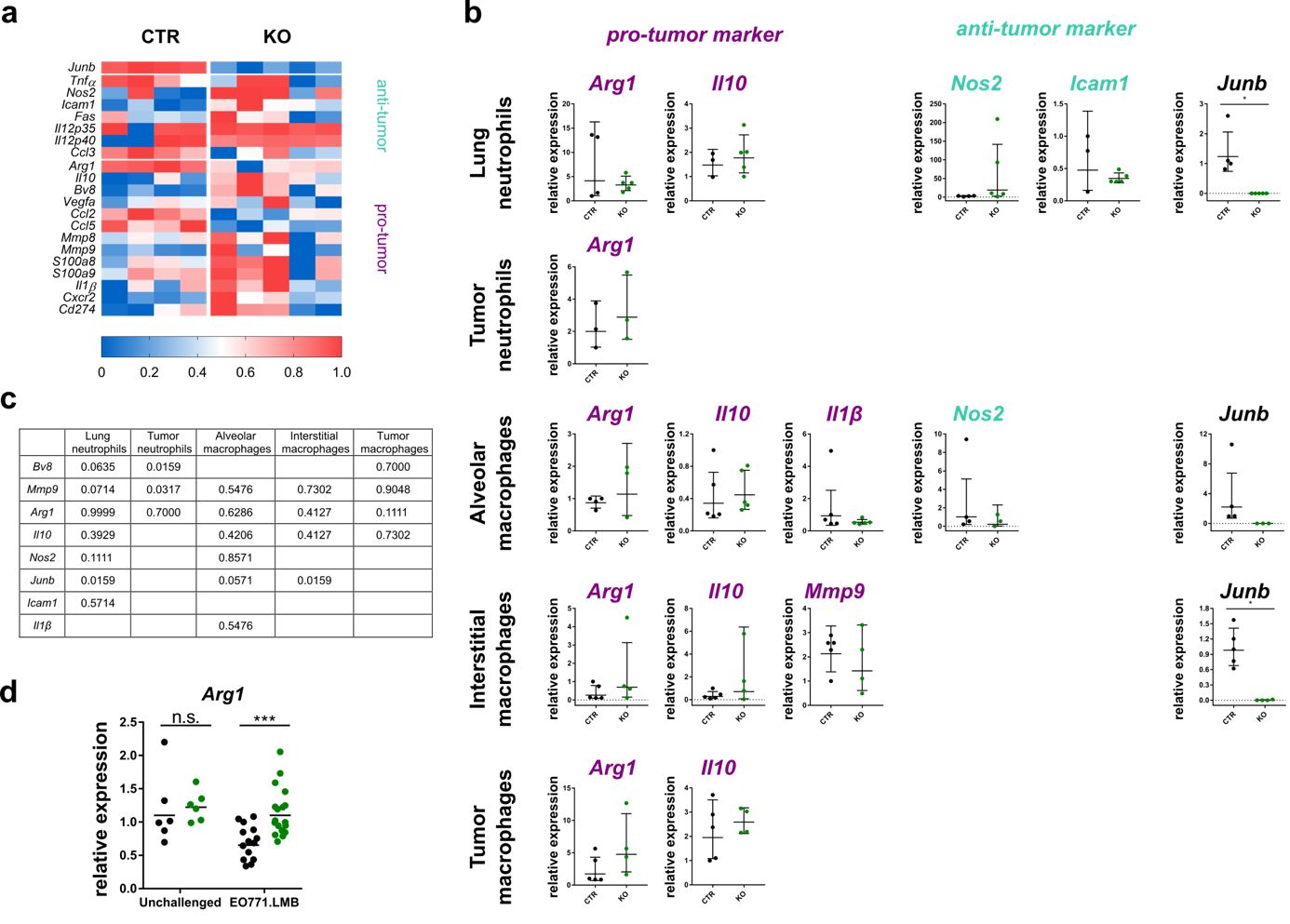
**

Supplementary Fig. 6

**
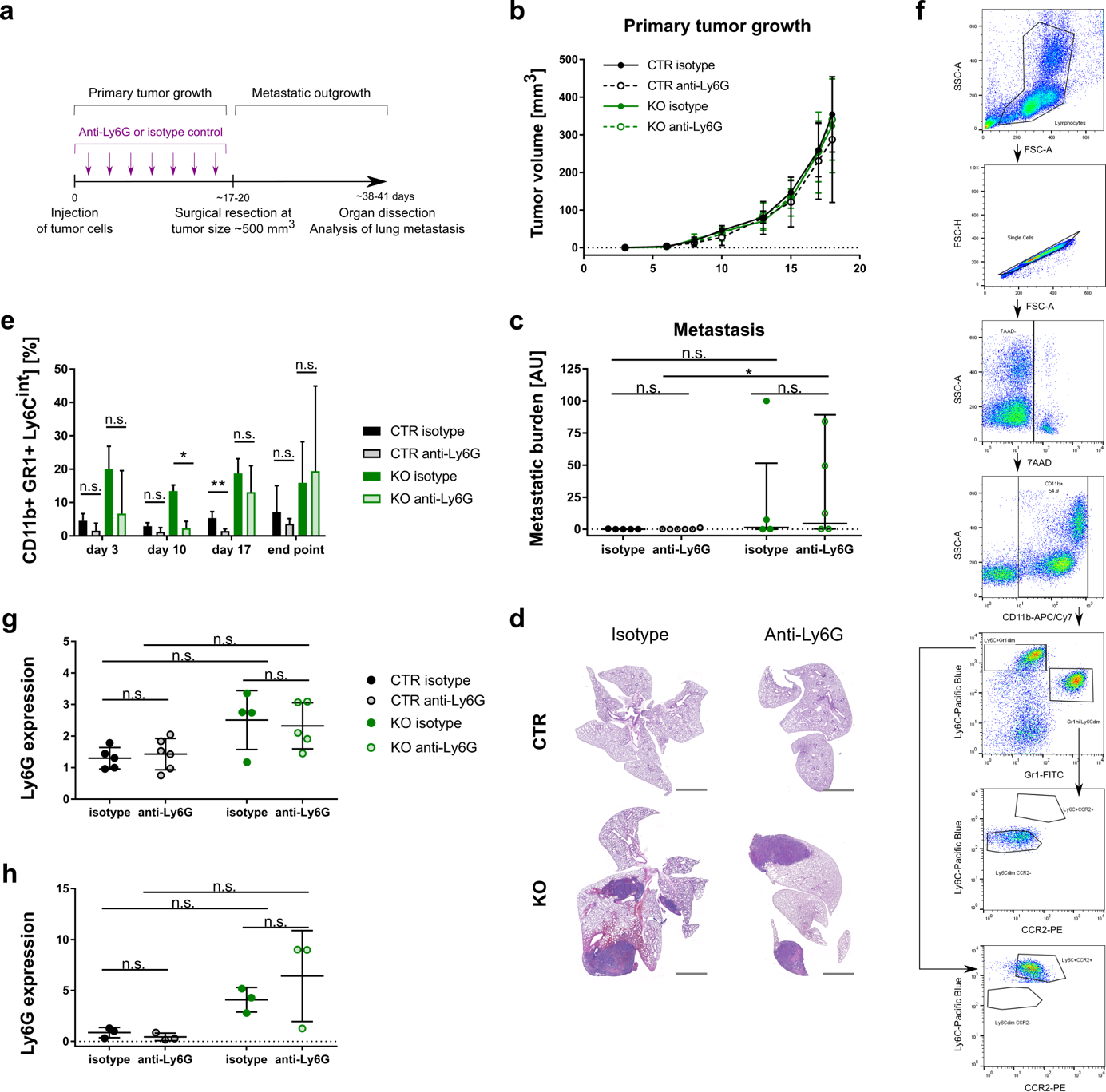
**

**Supplementary Table S1: Clinicopathological characteristics and JUNB expression in human breast cancer tissue microarray**

| **Variables** | **Overall Cohort** | **Analyz-able** | **Tumor** | | **Stroma** | |
| --- | --- | --- | --- | --- | --- | --- |
|  |  |  | **JUNB expression** | | **JUNB expression** | |
|  |  |  | **Positive**  **specimen** | **Mean H-score** $\boldsymbol{+}$**SD** | **Positive**  **specimen** | **Mean H-score** $\boldsymbol{+}$**SD** |
| **Overall samples,**  **n (%)** | 118  (100) | 33  (28) |  |  |  |  |
| **Subtype, n (%)** |  |  |  |  |  |  |
| IDC | 56 (47) | 22 (67) | 22 | 155±70 | 22 | 86±38 |
| ILC | 42 (36) | 6 (18) | 6 | 184±57 | 6 | 62±21 |
| DIVI | 11 (9) | 2 (6) | 2 | 217±37 | 2 | 99±14 |
| Others (1 x each MUC, MED, TUB) | 4 (3) | 3 (9) | 3 | 145±44 | 3 | 66±42 |
|  |  |  |  |  |  |  |
| **Receptor status** |  |  |  |  |  |  |
| ER+, Pr+ | 83 (70) | 19 (58) | 19 | 170±61 | 19 | 76±28 |
| Triple negative | 7 (6) | 4 (12) | 3 | 180±37 | 3 | 91±35 |
| Triple positive | 7 (6) | 3 (9) | 4 | 170±84 | 4 | 90±35 |
| Her2+ | 8 (7) | 4 (4) | 3 | 158±72 | 3 | 111±61 |
| ER+ | 5 (4) | 1 (3) | 1 | 125 | 1 | 61 |
| PR+ | 4 (3) |  | 1 | 170 | 1 | 92 |
| ER+, HER2+ | 2 (2) | 1 (3) | 1 | 31 | 1 | 21 |
| PR+, HER2+ | 2 (2) | 1 (3) | 1 | 62 | 1 | 73 |
|  |  |  |  |  |  |  |
| **Metastasis** | 8 (7) | 3 (9) | 3 | 210±54 | 3 | 95±27 |
|  |  | 5* (15) | 5* | 210±40 | 5* | 100±28 |

*2 samples included for which only one core could be evaluated

Supplementary Table S2: List of qPCR primers

| Gene | Forward/  reverse | Sequence | Efficiency | Reference | |
| --- | --- | --- | --- | --- | --- |
| *Arg1* | for | TTGGGTGGATGCTCACACTG | 2.02 | [1] | |
|  | rev | TTGCCCATGCAGATTCCC |  |  |  |
| *B2m* | for | CTCGGTGACCCTGGTCTTTC | 1.9 |  | |
|  | rev | TTGAGGGGTTTTCTGGATAGCA |  |  |  |
| *Bv8* | for | GCCCCGCTACTGCTACTTC | 2.2 |  | |
|  | rev | CCGCACTGAGAGTCCTTGTC |  |  |  |
| *Ccl2* | for | CGGCTGGAGCATCCACGTGTT | 2.02 | [2] | |
|  | rev | TAGCAGCAGGTGAGTGGGGC |  |  |  |
| *Ccl3* | for | TGTACCATGACACTCTGCAAC | 1.96 | [2] | |
|  | rev | CAACGATGAATTGGCGTGGAA |  |  |  |
| *Ccl5* | for | CCTCACCATATGGCTCGGACACC | 2.07 | [2] | |
|  | rev | GCGCGAGGGAGAGGTAGGCA |  |  |  |
| *Cd274* | for | GACCAGCTTTTGAAGGGAAATG | 1.88 | [3] | |
|  | rev | CTGGTTGATTTTGCGGTATGG |  |  |  |
| *Cxcr2* | for | CTTCCAGTTCAACCAGCCCT | 1.99 |  | |
|  | rev | CTTAATCCTGCAGTAGTTCTACGA |  |  |  |
| *Fas* | for | TGCTTGCTGGCTCACAGTTA | 2.0 |  | |
|  | rev | AGCAAAATGGGCCTCCTTGA |  |  |  |
| *Gfp reporter* | For | GCACTGACAATTCCGTGGTG | 1.87 |  | |
|  | rev | GAAGGTCCGCTGGATTGAGG |  |  |  |
| *Icam1* | for | ACCCACCCCGCAGGTCCAAT | 1.92 | [2] | |
|  | rev | CAGCCGAGGACCATACAGCACG |  |  |  |
| *Il10* | for | GCTCTTACTGACTGGCATGAG | 2.0 | PrimerBank ID 6754318a1 [4, 5] | |
|  | rev | CGCAGCTCTAGGAGCATGTG |  |  |  |
| *Il1β* | for | TTCAGGCAGGCAGTATCACTC | 1.99 | PrimerBank ID 118130747c2/  118130747c3 [4, 5] | |
|  | rev | CGGAGCCTGTAGTGCAGTTG |  |  |  |
| *Il12p35* | for | GCATGTGTCAATCACGCTACC | 1.96 |  | |
|  | rev | GAGACTGGAATGACCCTGGC |  |  |  |
| *Il12p40* | for | ACAAGACTTTCCTGAAGTGTGAAG | 1.91 | [6] | |
|  | rev | GCTCTTGATGTTGAACTTCAAGTCC |  |  |  |
| *Itgam (Cd11b)* | for | CATCCCCCTGCAAGTACCTC | 1.92 |  | |
|  | rev | GGGGGACAGTAGAAACAGCC |  |  |  |
| *Junb* | for | ACGCCGCCTGTGTCCCCCATCAA | 1.89 |  | |
|  | rev | CACTCGACAGCCCCGCGTTCTCAG |  |  |  |
| *Ly6g* | for | TTGTATTGGGGTCCCACCTG | 1.91 | [7] | |
|  | rev | CCAGAGCAACGCAAAATCCA |  |  |  |
| *mCherry* | for | GACCACCTACAAGGCCAAGAAG | 1.93 | [8] | |
|  | rev | AGGTGATGTCCAACTTGATGTTGA |  |  |  |
| *Mmp8* | for | CCAAGCATGTTCCCAGGAGT | 1.96 |  | |
|  | rev | ACTGAAGAAGAGGAAGAAGGAGTC |  |  |  |
| *Mmp9* | for | CTCTGCTGCCCCTTACCAG | 1.93 |  | |
|  | rev | TAGCGGTACAAGTATGCCTCTG |  |  |  |
| *Nos2* | for | AGAGTGAAAAGTCCAGCCGC | 1.93 |  | |
|  | rev | GAAGATCCCCAGGTGTTCCC |  |  |  |
| *Pecam1* | for | CAAAATAAAGACCCCCAGAACATGG | 1.92 |  | |
|  | rev | TAAGGGAGCCTTCCGTTCTCT |  |  |  |
| *Ppia* | for | AATTCATGTGCCAGGGTGGTG | 1.84 |  | |
|  | rev | TGCCTTCTTTCACCTTCCCAA |  |  |  |
| *Ptprc (CD45)* | for | GAGGTGTCTGATGGTGCAAG | 1.99 | [9] |  |
|  | rev | TGTATTCCACTAAAGCCTGATGAA |  |  |  |
| *S100a8* | for | TCCTTGCGATGGTGATAAAA | 1.96 | [10] |  |
|  | rev | GGCCAGAAGCTCTGCTACTC |  |  |  |
| *S100a9* | for | CACCCTGAGCAAGAAGGAAT | 1.99 | [10] |  |
|  | rev | TGTCATTTATGAGGGCTTCATTT |  |  |  |
| *Tnfα* | for | GTAGCCCACGTCGTAGCAAA | 1.89 |  |  |
|  | rev | TTGAGATCCATGCCGTTGGC |  |  |  |
| *Vegfa* | for | CTTGTTCAGAGCGGAGAAAGC | 1.91 | [2] |  |
|  | rev | ACATCTGCAAGTACGTTCGTT |  |  |  |
| *Vimentin* | for | AGCTGCTAACTACCAGGACACTATTG | 1.9 | [8] |  |
|  | rev | CGAAGGTGACGAGCCATCTC |  |  |  |

Supplementary Table S3: Antibodies used for immunohistochemistry and immunofluorescene

| Antibody (clone) | Antigen retrieval | Dilution | Catalogue number |
| --- | --- | --- | --- |
| Rabbit anti-JUNB (C37F9) | Citric buffer, pH6 | 1:200/1:500 | CST #3753 |
| Rat anti-CD31 (MEC 13.3) | ‑ | 1:100 | BD #550274 |
| Rabbit anti-CD31 | Tris/EDTA, pH9 | 1:200 | Abcam #ab28364 |
| Rat anti-Lyve-1 (ALY7) | Citric buffer, pH6 | 1:100 | Affymetrix #14-0443 |
| Rat anti-CD45 (30-F11) | Citric buffer, pH6 | 1:100 | Novus #NB100-77417 |
| Rabbit anti-mCherry | Proteinase K | 1:300 | Abcam #ab167453 |
| Syrian hamster Podoplanin (8.1.1) | Citric buffer, pH6 | 1:500 | Hybridoma Bank |
| Rat anti-Neutrophil (7/4) | Tris/EDTA, pH9 | 1:300 | Abcam #ab53457 |
| Chicken anti-GFP | Citric buffer, pH6 | 1:600 | Abcam #ab13970 |

**Supplementary methods**

**Neutrophil depletion *in vivo:***

For neutrophil depletion, mice were treated with neutrophil depleting antibody anti-Ly6G (InVivoPlus, clone 1A8, BioXCell, Lebanon, NH, USA) or respective rat IgG2α anti-trinitrophenol isotype control (InVivoPlus, clone 2A3, BioXCell) by i.p. injection of 100 µg three times a week during the initial phase of tumor growth until surgical removal of the primary tumor. Once a week, blood was collected from the tail vein for flow cytometric analysis of the neutrophil depletion efficiency and stored in EDTA-treated tubes until use (Microvette^®^, Sarstedt, Nümbrecht, Germany).

**Preparation of single-cell suspensions for Magnetic cell sorting (MACS) and FACS:**

To analyze immune cell populations from EO771.LMB mCherry-positive primary tumors, pre-metastatic lungs and spleens, mice were sacrificed and organs harvested when the tumor had reached a volume of approximately 500mm^3^. Organs were excised after perfusion with PBS and immediately placed on ice in PBS to prevent them from drying out. Lung and tumor tissues were digested after manual mincing with scissors in a mix of Liberase TM (0.52 Wunsch units/mL, Roche, Mannheim, Germany) and DNase I (25 µg/mL, Sigma, Steinheim, Germany) in serum-free DMEM (Sigma) for 30-45 min at 37°C and mild shaking. Digestion was stopped by adding 8% FCS/DMEM and undigested tissue fragments were removed by pipetting and passing the suspension through a moistened 100 µm nylon filter. Spleens were mechanically homogenized in 3% FCS/RPMI using the back of a plunger. After pelleting and erythrocyte lysis in ACK lysis buffer (150 mM NH_4_Cl, 10 mM KHCO_3_, 0.1 mM EDTA, pH 7.2-7.4) on ice, still remaining cell clumps were dissolved by careful pipetting and filtering through a 70 µm nylon filter in FACS buffer (2% (w/v) bovine serum albumin, 5 mM EDTA in PBS). After centrifugation, cell pellets were resuspended in FACS buffer and manually counted using a Neubauer chamber. Dead cells were excluded from counting by addition of trypan blue.

For flow cytometric analysis of the neutrophil depletion efficiency, blood stored in EDTA-treated collection tubes was incubated with ACK lysis buffer as described above. Subsequent to 3 min incubation on ice, lysis was stopped by adding FACS buffer. If the cell pellet remained red after centrifugation, treatment with ACK lysis buffer was repeated until all erythrocytes had been removed. The final white cell pellet was resuspended in FACS buffer and manually counted as described above.

In order to prevent activation of neutrophils, ACK lysis and all centrifugation steps of blood samples used for neutrophil isolation by MACS were performed at RT.

**Stimulation of T cells *ex vivo:***

Single cell suspensions of tumor, spleen und pre-metastatic lung samples were incubated with 1x Brefeldin A solution (from 1000x stock, Biolegend, San Diego, CA, USA, #420601) in T cell medium (DMEM, 10% FCS, 0.1% ß-Mercaptoethanol, 5 mM HEPES, 1% Penicillin-Streptomycin, 1% Sodium Pyruvate). Cells were stimulated with 10 ng/mL Phorbol 12-myristate 13-acetate (PMA, Cayman Chemical, Ann Arbor, MI, USA, # Cay10008014-1) and 1 µg/mL Ionomycin (Cayman Chemical # Cay11932-1) and incubated for 6 h at 37°C and 5% CO_2_ before proceeding with flow cytometric analysis of intracellular Interferon γ production.

**Isolation of Neutrophils by Magnetic Cell Sorting (MACS):**

Neutrophils were isolated from ACK lysis buffer-treated blood with the Neutrophil Isolation Kit (Miltenyi Biotech #130-097-658) and MS columns (Miltenyi Biotech #130-042-201) according to the manufacturer’s manual. Cell number of eluted neutrophils was determined manually as outlined above. Approximately 1x10^5^ cells were set aside for flow cytometric analysis of purity, whereas the remaining cells were pelleted. The cell pellet was lysed in 700 µL QIAzol® lysis reagent, snap frozen in liquid nitrogen and stored at -80°C until RNA isolation.

**Intracellular FACS staining and panels:**

Staining of intracellular antigens (Interferon γ) was conducted with the aid of the Transcription Factor Buffer Set (BD Biosciences #562574) according to the manufacturer’s manual. Following staining of dead cells with fixable viability dye and surface markers, cells were fixed in 1x Fix/Perm buffer obtained by diluting 4x Fix/Perm buffer with Diluent buffer. After 40 min at 4°C, cells were washed and permeabilized in 1x Perm/wash solution before incubating with intracellular antibodies (Interferon γ) diluted in 1x Perm/wash solution. Cells were washed in 1x Perm/wash solution subsequent to 40 min incubation at 4°C and resuspended in FACS buffer.

Compensation controls were generated by incubating UltraComp eBeads Compensation Beads (Thermo Fisher, Eugene, OR, USA, #1-2222-42) with 0.5 µL of the respective antibodies. Unstained single cell suspensions of primary tumors containing mCherry+ tumor cells were used to compensate for mCherry. Gates were drawn according to Fluorescence minus one controls.

For FACSorting, cells were defined as follows: neutrophils (mCherry- CD45+ CD3- B220- CD11b+ Ly6G+ Ly6C^int^), alveolar macrophages (mCherry- CD45+ CD3- B220- Ly6G- CD11b^low^ SiglecF^high^ CD11c^high^) and interstitial macrophages (mCherry- CD45+ CD3- B220- Ly6G- CD11b+ SiglecF- CD11c- F4/80+ Ly6C-).

General immune cell panel: CD45-Pacific Blue (clone 30-F11, Biolegend #103126, 1:100), CD11b-APC (clone M1/70, BD #553312, 1:200), CD4-BUV395 (clone RM4-5; BD #740208, 1:100), CD8-BV711 (clone 53-6-7, Biolegend #100759, 1:100), Ly6C-BV605 (clone HK1.4, Biolegend #128035, 1:100), Ly6G-FITC (clone 1A8, BD Biosciences #551460, 1:500), CD3-PE (clone 145-2C11, Biolegend #100308, 1:100), LIVE/DEAD™ Fixable Near-IR Dead Cell Stain Kit (Thermo Fisher # L34975).

Myeloid panel: CD45-BV785 (clone 30-F11, Biolegend #103126, 1:100), CD11b-APCFire750 (clone M1/70, Biolegend #101261, 1:200), CD3ε-PE (clone, 145-2C11, Biolegend #100306, 1:500), CD45R/B220-PE (clone RA3-6B2, BD #553089, 1:200), Ly6C-PerCP/Cy5.5 (clone HK1.4, Biolegend #128011, 1:100), Ly6G-FITC (clone 1A8, BD #551460, 1:500), CD11c-Pacific Blue (clone N418, Biolegend #117322, 1:100), SiglecF-BUV395 (clone E50-2440, BD #740280, 1:200), F4/80-APC (clone BM8, Biolegend #123115, 1:160), Fixable Viability Dye eFluor™ 506 (ebioscience, San Diego, CA, USA, #65-0866-14).

T cell activation panel: CD45-APC-Cy7 (clone 30-F11, Biolegend #103115, 1:100), CD3ε-FITC (clone 145-2C11, Biolegend #100306, 1:500), CD4-BUV395 (clone RM4-5, BD# 740208, 1:100), CD8-BV711 (clone 53-6.7, Biolegend #100759, 1:100), INFγ-eFluor450 (clone XMG1.2, Invitrogen, Carlsbad, CA, USA, #48-7311-80, 1:500), Fixable Viability Dye eFluor™ 506.

Neutrophil depletion efficiency: CD11b-APCFire750 (clone M1/70, Biolegend #101261, 1:200), Gr1-FITC (clone RB6-8C5, Biolegend #108405, 1:200), Ly6C-Pacific Blue (clone HK1.4, Biolegend #128013, 1:200), CCR2-PE (clone 475301, R&D, Minneapolis, MN, USA, #FAB5538P-025, 1:50), 7-AAD.

Neutrophil purity after isolation: CD11b-APCFire750 (clone M1/70, Biolegend #101261, 1:200), Ly6C-Pacific Blue (clone HK1.4, Biolegend #128013, 1:200), Ly6G-FITC (clone 1A8, BD Biosciences #551460, 1:500), 7-AAD.

**Hematoxylin eosin staining:**

Organs were fixed in paraformaldehyde for 24 h before transferring to 70% ethanol and subsequent paraffin embedding. Sections of 6 µm were deparaffinized and rehydrated in decreasing concentrations of ethanol. Sections were immersed in hematoxylin (Morphisto, Frankfurt am Main, Germany) for 1-3 min, rinsed in water for 10 min and counterstained with eosin (Morphisto) for 1 min, subsequently dehydrated in increasing concentrations of ethanol and mounted in Eukitt (O.Kindler, Bobingen, Germany).

**Tissue microarray:**

Human tissue microarray samples were obtained from the National Center for Tumor Disease (Institute of Pathology, University Hospital Heidelberg, Germany) in accordance with its regulations and the Declaration of Helsinki. Study approval was obtained by the ethics committee of the University of Heidelberg (S-206/2005). The tissue microarray consisted of 118 primary untreated breast tumors (TMA23-II). Classification of tumors was done according to the World Health Organization classification of tumors of the breast [11]. Estrogen-, progesteron and human epidermal growth factor receptor 2 (HER2/(neu)) receptor status of the tumors had been defined previously by immune histochemistry according to current guidelines recommendations from the American Society of Clinical Oncology/College of American Pathologists [12,13].

Samples were stained for JUNB (C37F9, CST, Danvers, MA, USA, 1:500) by immuno-histochemistry as previously described [14]. Imaging was performed on the Axio Scan.Z1 (Zeiss) and raw image files were adjusted for brightness, contrast and gamma using the Zen Blue software (Zeiss). The final scores for JUNB expression were established on one hand by multiplying the median scores of 3 independent observations for staining intensity (Score 1–3, concerning 1 = no staining and 3 = high intensity) and number of stained tumor cells (Score 1–4, concerning 1 = no stained cells, 2 < 33%, 33% ≤ 3 ≥ 66%, and 4 > 66% stained cells). Only specimen for which both cores could be evaluated were included. For automated scoring, the IHC stained TMA was scanned with the VENTANA DP 200 Slide Scanner (Roche Germany). The QuPath software (version v0.2.2) was utilized for semi-automatic quantification of the immunohistochemical staining [15]. In the first step, the TMA grid was inferred by the TMA dearrayer function, followed by manually excluding invalid samples as well as staining artefacts. Next, the staining vectors were automatically determined and the positive cell detection function was used to quantify the number of positive tumor or stromal cells for each sample, respectively. An H-score for the tumor tissue was calculated for each tissue core by following equation: H-score= ((1*[weakly stained tumor nuclei])+(2* *[moderately stained tumor nuclei])+(3**[strongly stained tumor nuclei]))/([total number of tumor nuclei]*100]). An adapted equation was used to calculate H-scores for stromal tissue. Statistical analysis was performed using GraphPad Prism 9.0.0 (GraphPad Software, San Diego, USA).

**References**

1. Sharda DR, Yu S, Ray M, Squadrito ML, De Palma M, Wynn TA, Morris SM, Jr., Hankey PA (2011) Regulation of macrophage arginase expression and tumor growth by the Ron receptor tyrosine kinase. J Immunol 187 (5): 2181-92. doi:10.4049/jimmunol.1003460

2. Shaul ME, Levy L, Sun J, Mishalian I, Singhal S, Kapoor V, Horng W, Fridlender G, Albelda SM, Fridlender ZG (2016) Tumor-associated neutrophils display a distinct N1 profile following TGFβ modulation: A transcriptomics analysis of pro- vs. antitumor TANs. Oncoimmunology 5 (11): e1232221. doi:10.1080/2162402x.2016.1232221

3. Barsoum IB, Smallwood CA, Siemens DR, Graham CH (2014) A mechanism of hypoxia-mediated escape from adaptive immunity in cancer cells. Cancer Res 74 (3): 665-74. doi:10.1158/0008-5472.Can-13-0992

4. Spandidos A, Wang X, Wang H, Dragnev S, Thurber T, Seed B (2008) A comprehensive collection of experimentally validated primers for Polymerase Chain Reaction quantitation of murine transcript abundance. BMC Genomics 9 (1): 633. doi:10.1186/1471-2164-9-633

5. Spandidos A, Wang X, Wang H, Seed B (2009) PrimerBank: a resource of human and mouse PCR primer pairs for gene expression detection and quantification. Nucleic Acids Research 38 (suppl_1): D792-D9. doi:10.1093/nar/gkp1005 %J Nucleic Acids Research

6. Fontana MF, Baccarella A, Pancholi N, Pufall MA, Herbert DBR, Kim CC (2015) JUNB is a key transcriptional modulator of macrophage activation. J Immunol 194 (1): 177-86. doi:10.4049/jimmunol.1401595

7. Denaës T, Lodder J, Chobert M-N, Ruiz I, Pawlotsky J-M, Lotersztajn S, Teixeira-Clerc F (2016) The Cannabinoid Receptor 2 Protects Against Alcoholic Liver Disease Via a Macrophage Autophagy-Dependent Pathway. Sci Rep 6: 28806. doi:10.1038/srep28806

8. Johnstone CN, Smith YE, Cao Y, Burrows AD, Cross RS, Ling X, Redvers RP, Doherty JP, Eckhardt BL, Natoli AL, Restall CM, Lucas E, Pearson HB, Deb S, Britt KL, Rizzitelli A, Li J, Harmey JH, Pouliot N, Anderson RL (2015) Functional and molecular characterisation of EO771.LMB tumours, a new C57BL/6-mouse-derived model of spontaneously metastatic mammary cancer. Dis Model Mech 8 (3): 237-51. doi:10.1242/dmm.017830

9. Takase HM, Itoh T, Ino S, Wang T, Koji T, Akira S, Takikawa Y, Miyajima A (2013) FGF7 is a functional niche signal required for stimulation of adult liver progenitor cells that support liver regeneration. Genes Dev 27 (2): 169-81. doi:10.1101/gad.204776.112

10. Fusella F, Seclì L, Busso E, Krepelova A, Moiso E, Rocca S, Conti L, Annaratone L, Rubinetto C, Mello-Grand M, Singh V, Chiorino G, Silengo L, Altruda F, Turco E, Morotti A, Oliviero S, Castellano I, Cavallo F, Provero P, Tarone G, Brancaccio M (2017) The IKK/NF-κB signaling pathway requires Morgana to drive breast cancer metastasis. Nature Communications 8 (1): 1636. doi:10.1038/s41467-017-01829-1

11. Lebeau A, Kriegsmann M, Burandt E, Sinn HP (2014) [Invasive breast cancer: the current WHO classification]. Pathologe 35 (1): 7-17. doi:10.1007/s00292-013-1841-7

12. Hammond ME, Hayes DF, Dowsett M, Allred DC, Hagerty KL, Badve S, Fitzgibbons PL, Francis G, Goldstein NS, Hayes M, Hicks DG, Lester S, Love R, Mangu PB, McShane L, Miller K, Osborne CK, Paik S, Perlmutter J, Rhodes A, Sasano H, Schwartz JN, Sweep FC, Taube S, Torlakovic EE, Valenstein P, Viale G, Visscher D, Wheeler T, Williams RB, Wittliff JL, Wolff AC, American Society of Clinical O, College of American P (2010) American Society of Clinical Oncology/College of American Pathologists guideline recommendations for immunohistochemical testing of estrogen and progesterone receptors in breast cancer (unabridged version). Arch Pathol Lab Med 134 (7): e48-72. doi:10.1043/1543-2165-134.7.e48

13. Wolff AC, Hammond ME, Hicks DG, Dowsett M, McShane LM, Allison KH, Allred DC, Bartlett JM, Bilous M, Fitzgibbons P, Hanna W, Jenkins RB, Mangu PB, Paik S, Perez EA, Press MF, Spears PA, Vance GH, Viale G, Hayes DF, American Society of Clinical O, College of American P (2014) Recommendations for human epidermal growth factor receptor 2 testing in breast cancer: American Society of Clinical Oncology/College of American Pathologists clinical practice guideline update. Arch Pathol Lab Med 138 (2): 241-56. doi:10.5858/arpa.2013-0953-SA

14. Schmitt K, Molfenter B, Laureano NK, Tawk B, Bieg M, Hostench XP, Weichenhan D, Ullrich ND, Shang V, Richter D, Stögbauer F, Schroeder L, de Bem Prunes B, Visioli F, Rados PV, Jou A, Plath M, Federspil PA, Thierauf J, Döscher J, Weissinger SE, Hoffmann TK, Wagner S, Wittekindt C, Ishaque N, Eils R, Klussmann JP, Holzinger D, Plass C, Abdollahi A, Freier K, Weichert W, Zaoui K, Hess J (2019) Somatic mutations and promotor methylation of the ryanodine receptor 2 is a common event in the pathogenesis of head and neck cancer. International journal of cancer 145 (12): 3299-310. doi:10.1002/ijc.32481

15. Bankhead P, Loughrey MB, Fernandez JA, Dombrowski Y, McArt DG, Dunne PD, McQuaid S, Gray RT, Murray LJ, Coleman HG, James JA, Salto-Tellez M, Hamilton PW (2017) QuPath: Open source software for digital pathology image analysis. Sci Rep 7 (1): 16878. doi:10.1038/s41598-017-17204-5
